# One Health as a moral dilemma: Towards a socially responsible zoonotic disease control

**DOI:** 10.1111/zph.12536

**Published:** 2018-11-02

**Authors:** Joost van Herten, Bernice Bovenkerk, Marcel Verweij

**Affiliations:** ^1^ Department of Philosophy Wageningen University and Research Wageningen The Netherlands; ^2^ Royal Veterinary Association of The Netherlands Houten The Netherlands

**Keywords:** boundary object, ethics, health concepts, moral dilemma, moral status, One Health, zoonotic disease control

## Abstract

During the last decade, the concept of One Health has become the international standard for zoonotic disease control. This call for transdisciplinary collaboration between professionals in human, animal and environmental health has produced several successes in zoonotic disease control, surveillance and research. Despite the lack of a clear definition, a shared agenda or institutional governance, One Health has proven to be a fruitful idea. Due to its ambiguity, the One Health concept functions as a boundary object: by leaving room for interpretation to fit different purposes, it facilitates cooperation. In many cases, this results in the promotion of health of humans, animals and the environment. However, there are also situations in which this mutual benefit of a One Health approach is not that evident, for instance, when healthy animals are culled to protect public health. Although such a strategy could well be part of a One Health approach, it is hard to understand how this contributes to the health of concerning animals. Consequently, these practices often lead to public debate. This raises questions on how we should understand the One Health concept in zoonotic disease control. Is it really about equally improving the health of humans, animals and the environment and is this even possible? Or is it ultimately just public health that counts? In cases of conflict between different values, the lack of a universal definition of the One Health concept contributes to this complexity. Although boundary objects have many positive aspects, in the context of One Health and zoonotic disease control, this conception seems to conceal underlying normative differences. To address moral dilemmas related to a One Health approach in zoonotic disease control, it is important to reflect on moral status and the meaning of health for humans, animals and the environment.

**Impacts**

- Zoonotic disease control measures following the One Health concept can create moral dilemmas
- Current interpretations of One Health conceal normative differences in case of value conflicts; therefore, One Health needs a corresponding ethical framework.
- This ethic of One Health starts with discussing the moral status of animals and the environment and an appropriate definition of health. To understand health as “resilience” provides opportunities to improve the health of humans, animals and the environment by means of prevention rather than cure. In case of value conflicts, ethical principles, like VandeVeer's “two factor egalitarianism”, can offer policymakers moral guidance.

## 1 INTRODUCTION

In a globalized world where human activity has a devastating impact on ecosystems essential to human and animal life, an interdisciplinary, collaborative strategy to attain optimal health for people, animals and the environment is indispensable. Such a One Health approach is currently considered the worldwide standard to combat epidemic zoonotic threats like influenza, SARS or Ebola (FAO‐OIE‐WHO, 2010). An important reason for this success is that the One Health concept acts as a boundary object (Cassidy, 2016; Leboeuf, 2011). Star and Griesemer (1989) define a boundary object as a multi‐interpretable concept, that is, “both plastic enough to adapt to the local needs and the constraints of the several parties employing them, yet robust enough to maintain a common identity across sites”. The power of boundary objects is that they enable people with different perspectives to collaborate, in case of One Health, to promote health. This results in a number of successful One Health practices, for instance, in combatting zoonotic diseases like avian influenza and rabies (Gibbs, 2014).

Despite these achievements, several authors have criticized the One Health concept on issues of support and implementation (Okello, Bardosh, Smith, & Welburn, 2014; Stephen & Karesh, 2014). More recently, others have debated that a One Health approach in zoonotic disease control can also cause more fundamental moral dilemmas (Rock & Degeling, 2015; Verweij & Bovenkerk, 2016). This is because in zoonotic disease control there can be conflicts of interests, for instance, between public health institutions and the food industry. Furthermore, certain One Health strategies lead to the culling of healthy animals. If One Health implies that besides the health of humans, the health of animals and the environment should be promoted as well, this requires justification (Degeling, Lederman, & Rock, 2016).

However, current interpretations of the One Health concept neither in literature nor in policy documents provide normative guidance to address these moral dilemmas. Although recent standards like COHERE improve the quality of One Health research and the integration of all domains (Davis et al., 2017), there is still little attention for ethical issues. Lee and Brumme (2013) argue that a possible way forward could be to develop a widely supported operational definition.

We claim that, for two important reasons, this will not overcome the moral dilemmas that originate from One Health strategies in zoonotic disease control. Firstly, it will be complicated to reach consensus on the overall goal of a One Health approach because of underlying moral differences. Secondly, to impose a universal definition can obstruct One Health’s function as a boundary object to facilitate cooperation.

To be more than just a call for collaboration and to address the moral dilemmas that can arise in zoonotic disease control, the One Health concept needs a corresponding ethical framework. To start the discussion on such an ethic of One Health, we think it is necessary to first formulate some normative starting points about the moral status of humans, animals and the environment. Thereafter, we argue that this also implies a health concept that can be used for all elements within the One Health framework and we propose “resilience” as a fruitful option in this respect.

## 2 THE MERITS AND CRITICISM OF ONE HEALTH

Many health professionals think the One Health concept is crucial to “win the disease battles of the 21st century while ensuring the biological integrity of the planet for future generations” (Cook, Karesh, & Osofsky, 2004). One Health offered global institutions like the World Health Organization (WHO), the Food and Agricultural Organization of the United Nations (FAO) and the World Organization for Animal Health (OIE), an all‐inclusive approach to reduce conflicts, defend their legitimacy and facilitate commitment for collaboration (Chien, 2013). A shared statement confirmed their partnership to address health risks at the human–animal–ecosystem interface (FAO‐OIE‐WHO, 2010). This shows that One Health can facilitate partnerships and promote interdisciplinary collaboration despite possible conflicts of interest.^1^ 1The core activity of WHO is global public health, OIE is focussed on animal health and welfare, and the main interest of FAO is food security. Zoonotic disease control can lead to conflicts between, for example, animal welfare and global public health.


In the meantime, the One Health approach has also produced actual results in infectious disease control, like the Dutch policy to combat antimicrobial resistance in humans as well as in animals. In 2009, the Dutch government took strict measures to reduce the use of antibiotics in animals to protect public health. These actions were framed as a One Health policy and included recording and benchmarking of antibiotic use on farms, benchmarking of the prescribing patterns of veterinarians, strengthening the role of veterinarians, taking measures to improve animal health and promoting prudent use in line with official reduction targets. This public–private cooperation has resulted in a significant reduction of 64% in the use of antibiotics in animals in the Netherlands from 2009–2016 (Netherlands Veterinary Medicines Institute, 2017). The Food and Veterinary Office of the European Commission concluded the Netherlands showed it was possible to reduce the use of antibiotics in animals and associated antimicrobial resistance, while safeguarding animal health and welfare^2^ 2In 2016, the Dutch Council on Animal Affairs addressed this issue in its advice on the effects and perspectives of antimicrobial reduction policies in animal husbandry. The council concluded that because of a lack of data, an objective assessment of suspected animal welfare issues was difficult. Therefore, a causal relation between antimicrobial reduction policies and animal welfare problems could not be determined (Council on Animal Affairs, 2016). and the economic viability of producers, and avoiding an excessively legislative approach (FVO, 2017).

One Health approach strategies are often more effective than regular public health and disease control measures and also more efficient, because services responsible for human, animal and environmental health can share costs. In 2012, the World Bank concluded that this could add up to a 15% reduction for a global surveillance and disease control system. Due to investments in One Health systems, yearly financial benefits could exceed initial costs 10‐fold. Apart from the financial gains, combatting zoonotic disease with a One Health strategy would substantially improve public health, food safety and food security as well (York et al., 2012).

Despite these merits of a One Health approach, in recent years, several critical notes have been published too. First of all, some authors noticed that the involvement of human medicine in the discussions on One Health was poor (Häsler et al., 2012). Secondly, the lack of involvement of stakeholders from the environmental sector is worrying. Because important environmental determinants of health, like climate change and pollution are then underexposed despite their impact on human and animal health and welfare (Stephen & Karesh, 2014). Others point out that there is no shared One Health agenda and global health governance by existing institutions is failing (Lee & Brumme, 2013). Finally, there are also worries about the implementation of One Health with respect to national ownership and funding, certainly in the developing countries (Okello et al., 2014).^3^ 3This study shows that in countries like Nigeria, Tanzania and Uganda it generally takes longer to implement One Health strategies, often as a result of the wide institutional and policy changes required. The authors conclude that: “while the ‘goodwill’ is certainly there, the reality of planning, executing and budgeting for joint interventions—particularly at the national or regional level—proves in many cases more difficult than first thought”.


## 3 MORAL DILEMMAS OF ONE HEALTH

The application of the One Health concept in zoonotic disease control also raises important ethical questions (Degeling et al., 2016; Lederman, 2016; Rock & Degeling, 2015). If One Health implies we should promote the health of humans as well as the health of animals and the environment, how does this work out in the case of zoonotic disease control?

An interesting example is the search and destroy policy for multi‐resistant bacteria on pig farms in Norway. Multi‐resistant bacteria like livestock‐associated methicillin‐resistant *Staphylococcus aureus* (LA‐MRSA) can be a threat to human health (Fitzgerald, 2012). Especially, after introduction in hospitals, there is a risk that LA‐MRSA causes serious and sometimes untreatable infections in susceptible patients. For this reason, in many countries, pig farmers and veterinarians, who have a higher risk of introducing LA‐MRSA into hospitals, are subjected to strict hygiene and quarantine measures to prevent nosocomial infections in vulnerable patients.

LA‐MRSA is widespread amongst pigs and cattle in Europe, but the prevalence varies greatly between countries (European Food Safety Authority, European Centre for Disease Prevention, & Control, 2017). Because in Norway LA‐MRSA prevalence in pigs is very low, Norwegian authorities choose to cull all pigs on a farm when LA‐MRSA is detected. Prevention of LA‐MRSA introduction on pig farms can be more cost‐effective than implementing expensive preventive measures in hospitals (Höjgård et al., 2015). However, in the Netherlands, such a policy would be devastating for the pig industry because the prevalence of LA‐MRSA is more than 70% (Broens, Graat, Van Der Wolf, Van De Giessen, & De Jong, 2011).

Until recently, LA‐MRSA was considered a serious health threat in hospitals in the Netherlands. But since it has become clear that LA‐MRSA does not spread as easily in hospitals as was earlier suspected, LA‐MRSA policies in Dutch hospitals are mitigated (Meekelenkamp, Schneeberger, Hermans, Janssen, & Robben, 2017). This raises questions about the proportionality of LA‐MRSA policies in Norway. Moreover, research has shown that LA‐MRSA infections on pig farms in Norway were introduced by farm workers (Grøntvedt, Elstrøm, Stegger, Skov, & Skytt Andersen, 2016). This implies that the health of pigs, usually not affected by LA‐MRSA but culled after detection, is maybe more at risk than the other way around. The discrepancy between Norway and the Netherlands might be justified on the basis of differences of LA‐MRSA prevalence, cost‐effectiveness and risk perception. However, it is difficult to see how pigs in Norway benefit from this One Health approach.

Apparently, the recognition that human, animal and environmental health are intertwined does not necessarily imply that they are all of similar weight. It often seems as if animal and environmental health are only deemed worthy of protection as long as they contribute to human health. But if this is the case, One Health is in fact nothing more than just another label for protecting public health. If One Health should be regarded as the paradigm shift that some authors envision, safeguarding human health is not enough. Maintenance and improvement of animal health and ecosystem functioning are also primary goals of One Health, with their own inherent value independent from their impact on human health (Barrett & Osofsky, 2013). Degeling et al. (2016) therefore suggest to regard health as a universal good: a necessary condition for a flourishing life which is shared between species, ecosystems and future generations. Contrary to public goods—which only apply to humans—this means that animals and the environment are considered recipients as well. Just like in public health policies, where there is attention for distributive aspects of health in human populations, the One Health paradigm forces us to think about a fair distribution of health between humans, animals and environment.

## 4 CONCEPTUAL CLARITY

The advantage of regarding One Health as a boundary object is that flexibility in interpretation facilitates cooperation and makes the concept applicable for multiple purposes. However, ambiguity about One Health and hence about how One Health strategies in zoonotic disease control should be shaped contributes to the complexity in case of value conflicts. If it is not clear beforehand what the normative starting points of a One Health approach are, different parties can disagree about the expectations and results for the health of humans, animals and the environment.

In literature and in policy documents, many definitions of the One Health concept can be found (Gibbs, 2014). The American Veterinary Association defines One Health as “an integrative effort of multiple disciplines working locally, nationally and globally to attain optimal health for people, animals and the environment” (AVMA, 2008). While the Food and Agricultural Organisation speaks of “a collaborative, international, multidisciplinary mechanism to address threats and reduce risks of detrimental infectious diseases at the human–animal–ecosystem interface” (FAO, 2012). Finally, the One Health Initiative defines One Health as “a worldwide strategy for expanding interdisciplinary collaborations and communications in all aspects of health care for humans, animals and the environment” (Kahn, Kaplan, & Monath, 2012).

Cassidy points out that the existing definitions are often strikingly broad. They all promote interdisciplinary collaboration but do not specify who should be collaborating with whom, on what and how (Cassidy, 2016). That is why some authors stress the need for an agreed operational definition (Lee & Brumme, 2013). Others, like Leboeuf (2011), say the broadness of the concept makes it possible to act as an umbrella under which One Health actors can articulate slightly different visions while working together. Chien (2013) also believes that One Health is sufficiently concrete and flexible enough to facilitate collaboration. However, she does think that its vagueness also allows conflicting interpretations to coexist. This can hinder a paradigm shift in disease policies and tolerates a situation where health professionals keep practising within the dominant technical/biomedical framework without converting to the holistic One Health perspective (Chien, 2013). In our view, further conceptual analysis of One Health is indeed necessary to transcend the level of a mere collaboration tool. When the concept of One Health is elaborated as we propose, it can create opportunities to actually contribute to the health of all.

In her historic analysis of zoonotic disease control in the Netherlands (1898–2001), Haalboom argued that interdisciplinary collaboration between veterinary and human medicine was never the issue. Veterinarians and human doctors have cooperated to combat zoonotic diseases long before One Health became fashionable (Haalboom, 2017). In this view, the urgent call for collaboration will only partially address the risk of zoonotic diseases for public health. She concludes that the real problem in zoonotic disease control is an underlying conflict of interests. The most prominent in this perspective is the clash between public health and economy. Until the Q fever outbreak (2007–2012^4^ 4During the Q fever outbreak that struck the Netherlands from 2007–2012, the Dutch government decided to cull 50,000 pregnant goats to stop the disease that until then infected 4,000 people and (with hindsight) caused the death of 74 persons. A vaccination strategy was carried out since the end of 2008, but because a positive effect was not immediately visible and the numbers of human casualties continued to rise; in 2009, the Dutch government switched to culling.) the food industry proved to be very successful in promoting economic interests and delayed an effective response at the expense of public health (Haalboom, 2017).

Public health can also conflict with the health and welfare of animals. Think for instance of the case of culling healthy animals in a zoonotic disease outbreak. Lederman argues that culling as a public health measure can only be justified if there is enough evidence that such an intervention is (cost)effective and socially accepted, but in practice, this is not always the case (Lederman, 2016). A different interpretation of One Health could potentially replace standard disease control measures like culling, as it provides reasons to extend ethical consideration about public health policies and corresponding economic decision‐making processes beyond protecting short‐term human interests (Degeling et al., 2016). In our perspective, a One Health strategy should imply preventing zoonotic disease outbreaks by investing in the health of animals and the environment. This may also be helpful to avoid some apparent conflicts between public health and the health of animals.

Applying the One Health concept in zoonotic disease control is not morally neutral (Nieuwland & Meijboom, 2015). However, the current interpretation of the One Health concept in literature and policy documents lacks normative guidance for health professionals, institutions and governments in case of moral dilemmas. Building on this, we believe a One Health ethic first requires a basic foundation that acknowledges the moral standing of animals and the environment. Current conceptions of One Health are implicit about the question whether or not animals and the environment have independent moral standing at all. They leave room for interpretations that consider the health of animals and the environment as only of instrumental value for humans. Secondly, it is important to reflect on a possible concept of health that is appropriate within the One Health framework. For One Health to act as a boundary object, it may not be necessary to define the concept of health any further. But we think it is essential to address moral dilemmas of One Health strategies in zoonotic disease control. Clarity about a concept of health, that is suitable for humans, animals and the environment, enables us to reach a better understanding about our One Health goals.

## 5 THE VALUE OF MORAL STATUS OF HUMANS, ANIMALS AND THE ENVIRONMENT

It is clear that the health of animals and the environment is essential for humans. We know, for instance, that over the last 20 years more than 70% of all emerging infection diseases in humans are zoonotic of origin (Taylor, Latham, & Woolhouse, 2001). But is animal or environmental health also important in itself? This question is only meaningful when we attribute animals and the environment some kind of moral status. In animal and environmental ethics, there is an extensive debate about this topic.

To have moral status implies, being a member of our moral community, and therefore, one’s interests should be taken into account. In this context, Gruen has introduced the term moral considerability: “to say that a being deserves moral consideration is to say that there is a moral claim that this being has on those who can recognize such claims. A morally considerable being is a being who can be wronged in a morally relevant sense” (Gruen, 2014). In her opinion, this means a being is either morally considerable or it is not. She distinguishes this notion from moral significance, which in contrast to moral considerability can be a matter of degree. Moral significance indicates how we should assess and adjudicate different interests of morally considerable beings in situations of conflict (Gruen, 2014).

The perspective on moral considerability and corresponding moral significance is strongly influenced by the normative theoretical framework that is used (Bovenkerk & Meijboom, 2012).

Singer, for instance, thinks that the capacity to experience pain or pleasure (sentience) is the one and only relevant feature to attribute moral considerability (Singer, 1975). For Regan, entities are morally considerable when they are a subject‐of‐a‐life, which means that they have cognitive capacities such as beliefs, desires, memory, intentions and a sense of time and future, in other words that they can experience life subjectively (Regan, 2004). Other philosophers emphasize the importance of external properties, like our specific relations with animals that influence our obligations towards them (Palmer, 2010).

In case of conflict, a utilitarian, like Singer, would base his judgement on maximization of the satisfaction of preferences (or interests) of all morally relevant beings involved. If the culling of healthy animals would stop the spread of an infectious disease that could potentially infect many other animals (and possibly humans in case of a zoonotic disease), Singer thinks this is justified (Singer & Dawn, 2004). Regan would strongly reject such practices. On the basis of respect for the inherent value of all subjects‐of‐a‐life, he believes that no individual animal can be sacrificed for the good of the whole.

Some philosophers have tried to extend moral status beyond humans and animals. Aldo Leopold, for instance, argued that moral concern should extend from humans to: “soils, waters, plants and animals, or collectively: the land” (Leopold, 1949). In this perspective, the survival of an ecosystem as a whole is of more importance than the fate of individual living beings like humans, animals or plants which are part of this ecosystem. To underpin his claim for the moral relevance of ecosystems, Leopold explained that they could be harmed by human activities in a similar way that a disease could harm a human being (Leopold, 1949).

In most societies, there is a plurality of views on the moral considerability and significance of animals and the environment. From the results of a large survey in the Netherlands about people's convictions on the moral status of animals in relation to the culling of healthy animals in an animal disease outbreak, Cohen, Brom, and Stassen (2012) distinguished two main categories: those who consider humans superior to animals and those who think human and animal interests should be taken into account equally. Most of the former categories have no problems with the culling of healthy animals during an animal disease epidemic, while many of the latter disagree with such policies. When the reason for culling healthy animals was protection of human life, 39% of the people with an egalitarian viewpoint and 19% of the people who think humans are superior rejected this (Cohen et al., 2012). This shows that moral convictions have a strong influence on the perceived acceptability of certain disease control measures. However, it does not imply that if people consider themselves superior to animals, they all think that animals are not morally considerable. Even for some of those people culling is morally problematic. Moreover, the moral significance that people attribute to animals is case dependent. In cases where human health is at risk, most people justify the culling of healthy animals. In situations where there is no danger that humans become infected, culling is less accepted. Which disease control measures are justified in a One Health strategy to combat zoonotic diseases is therefore strongly dependent on the normative presuppositions people have.

We acknowledge that in our society moral values, norms, ideals, duties and virtues are in general irreducibly diverse. From this, one could argue that moral values are only relative to a person’s individual and cultural background or to certain circumstances. Moral relativists claim there is no moral truth nor an objective moral standard to decide what is right and wrong; what is right for me does not have to be right for you. On the contrary, moral pluralism acknowledges there can be conflicts in values, but this does not mean we cannot criticize each other’s moral viewpoints. This theory accepts that in case of two or more valid moral positions there is no single overarching principle to judge what is the right thing to do (Wolf, 1992). Yet, moral pluralists strive to make reasonable choices between conflicting moral values, to be action guiding in moral dilemmas. Although moral values are not beforehand overriding, under certain conditions some values can be more important than others (Kekes, 1993). Even if several conflicting positions can be deemed valid within moral pluralism, it does not follow that no wrong positions can be determined. Moral judgements are justified in a deliberative process searching for coherence between intuitions, moral values and principles and empirical facts: a reflective equilibrium. With this in mind, we propose to address value conflicts in zoonotic disease control in a similar manner.

In a One Health perspective, the principle of “two factor egalitarianism” that VanDeVeer (1979) introduced, could be useful to tackle conflicts of interests between humans and animals. VanDeVeer suggests that in promoting overall utility, a difference should be made in the level of importance of interests of humans and animals. In his theory, peripheral interests of humans do not prevail over basic interests of animals. But in the case of a conflict between basic interests, the interests of humans trump those of animals. VanDeVeer (1979) justifies this by arguing that “the interests of beings with more complex psychological capacities deserve greater weight than those with lesser capacities”. This implies that the harm that is caused by dying is in general greater for humans than it is for animals^5^ 5VandeVeer considers that the “opportunity costs” of dying are far greater for humans than for animals. This notion is derived from economic theory and indicates that in achieving one goal, the cost of doing so can be thought of as opportunities thereby forgone, goods and satisfactions that may not be obtained but which could have been if one's capital or effort were employed in other ways.. In case of zoonotic disease control, this implies that culling animals is only justified when basic interests of humans, like an interest in life or not suffer severely, are at stake. It may be questionable whether this principle will be applicable to all moral dilemmas in zoonotic disease control. Besides that, it is not entirely clear how environmental interests should be weighted within VanDeVeer’s method. However, we believe VanDeVeer’s principle can be helpful as a starting point by establishing that basic animal and environmental interests cannot be overridden by peripheral human interests. Certainly, in a pluralist society, there will be different opinions about what should be regarded as a peripheral or a basic interest. It can be debated, for instance, if in Western societies eating meat should be considered a peripheral interest or not.

Nevertheless, we think in many situations, it will be possible to reach consensus by considering whether or not certain human interests are strong enough to violate basic animal and environmental interests. In our opinion, a One Health strategy in zoonotic disease control entails that basic animal interests can only be overridden if there are no other reasonable alternatives to protect human health, like vaccination. Of course, the costs and the effectiveness of possible alternatives should be taken into account as well. However, economic reasons alone cannot justify culling as a disease control measure.

## 6 HEALTH FOR HUMANS, ANIMALS AND THE ENVIRONMENT

To clarify the normative assumptions in One Health, besides reflections on the issue of moral status, we also need to consider which concept of health is most suitable. If we define our ideas about health more specifically, this can give us guidance in determining what we strive for if we want to achieve a better health for humans, animals and the environment. To our opinion, an appropriate concept of health should fulfil at least two requirements: (a) it should be separately applicable to humans, as well as to animals and the environment and (b) supportive to the idea of health of the system as a whole.

In human medicine, the concept of health has evolved over time. In 1946, the WHO defined health as a complete state of physical, mental and social wellbeing, not merely absence of disease or infirmity (WHO, 1946). Later, Boorse explained health as a condition of statistically normal biological functioning and therefore as absence of disease (Boorse, 1977). More recently, the focus is on definitions that see health as instrumental to achieve other goals in life (Nordenfelt, 1993). Finally, Huber et al. refer to health as the ability to adapt and self‐manage in the face of social, physical and emotional challenges (Huber et al., 2011). Nevertheless, to date, there is no universally agreed definition for human health.

In contrast to the extensive literature available on human health, there is much less scientific debate about the concept of animal health (Gunnarsson, 2006). In veterinary medicine, animal health is commonly interpreted in the tradition of Boorse’ absence of disease symptoms or as normality in biological functioning. Webster, for instance, refers to health as normality in posture, movement, alertness and appetite (Webster, 1987). Others state that animal health is the result of biological, social and environmental determinants that interact to affect the capacity to cope with change (Stephen, 2014). Some definitions include productivity as a parameter of animal health (Blood & Studdert, 1999). But paradoxically, animals that have been strongly selected for high productivity seem to be more at risk of behavioural, physiological and immunological problems (Rauw, Kanis, Noordhuizen‐Stassen, & Grommers, 1998). Few authors, like Nordenfelt, have tried to extend the concept of health from humans to animals. For him, animal health is instrumental to attain vital goals for an individual animal, like minimal animal welfare (Nordenfelt, 2006). However, up to now, there is also no consensus on a comprehensive definition for animal health.

Likewise, the concept of health is used to describe the functioning of an ecosystem^6^ 6An ecosystem can be defined as a complex of living organisms, their physical environment, and all their interrelationships in a particular unit of space (“ecosystem—Britannica Academic,” n.d.).. Lu, for instance, sees ecosystem health as “the status and potential of an ecosystem to maintain its organizational structure, its vigour of function and resilience under stress, and to continuously provide quality ecosystem services for present and future generations in perpetuity” (Lu et al., 2015). Health is then an indicator that is related to the deliverance of ecosystem services, like clean drinking water and fertile soil, for humans. Certainly in case of ecosystem health, most accounts are strictly anthropocentric. The more fundamental question is how to understand ecosystem health when we have to accept that ecosystems are dynamic and species come and go. In that case, a good starting point might be to strive for a situation of dynamic equilibrium. A concept that captures this aspect is “resilience”, defined as the capacity or ability of an individual or a system to react to an external force and to maintain or return to a state of equilibrium (Bhamra, Dani, & Burnard, 2011). The idea of resilience can be categorized within theories of health as some kind of balance (Lerner & Berg, 2015). According to Döring et al. (2015) resilience is applicable as a measurable criterion for health for all components of our ecosystem: soil, plants, animals and humans. Furthermore, resilience is also relevant for the ecosystem as a whole. This connection can, for example, be seen in relation to biodiversity. A monoculture of crops, as well as a diminished genetic diversity in pure bred animals or inbred human populations, often show a higher vulnerability to diseases. References to a certain form of resilience are found in health definitions for humans, animals and ecosystems (Huber et al., 2011; Lu et al., 2015; Stephen, 2014). Lerner and Berg (2015) claim that within a One Health framework health can be defined at least on three different levels: individual, population and ecosystem level. In our opinion, resilience is a meaningful concept to describe health at all of these levels.

It is clear that there are many conceptions of health with very different implications. Therefore, Haverkamp et al. propose to consider health concepts as a sort of family in which each concept has a slightly different descriptive and evaluative dimension and is applicable in a different context. In this way, they function as a toolbox to reflect on the meaning of health in specific health practices (Haverkamp, Bovenkerk, & Verweij, 2018). In the context of zoonotic disease control, this still leaves room to either choose a narrow account of health, like absence of disease, or a broader definition such as resilience. In general, it can be said that the thicker the concept of human health is defined, the more difficult it is to apply to animals or the environment. To regard health as the ability to adapt and self‐manage in the face of social, physical and emotional challenges is perhaps too ambitious for many domesticated animals. The restrictions of these animals related to their use by humans make this definition unrealistic. It is also hard to imagine how this conception can be applied to ecosystems.

To a certain degree, it is possible to assess absence of disease objectively in individual humans and animals. At population level, this perspective on health can be translated in epidemiological statistics or morbidity and mortality rates. Nevertheless, to determine ecosystem health in terms of absence of disease can be problematic. After all, pathogens like bacteria and viruses are an essential part of ecosystems. In the context of a One Health strategy in zoonotic disease control, it would therefore be more realistic to consider pathogens as something to work with rather than against (Hinchliffe, 2015). Until now, only two infectious diseases have been successfully eradicated on a global scale: Smallpox and Rinderpest. Actually, even these two viruses still exist because samples are stored in highly secured laboratories for possible vaccine production in case of disease re‐emergence. In many other cases, pathogen eradication has proven to be very difficult, certainly when there are non‐human reservoirs (Aylward, Hennessey, Zagaria, Olivé, & Cochi, 2000).

In addition, most recent pandemics of zoonotic origin and they often emerge by ecological, behavioural or socio‐economic changes, induced by human action (Morse et al., 2012). Loss of biodiversity, for instance, is one of the factors known to influence pathogen transmission and disease incidence. There are indications that preserving ecosystems and their biodiversity can reduce prevalence of infectious diseases in humans (Keesing et al., 2010). In case of Lyme disease and West Nile Virus, for instance, it appears that loss of biodiversity can promote the number of the host species for these pathogens. This is because they seem more resistant to factors that reduce biodiversity than other species (Keesing et al., 2010). From a One Health perspective, this implies that by promoting biodiversity in ecosystems, animal and human health are served as well. Biodiversity is considered a critical part of ecosystem resilience (Folke et al., 2004).

Moreover, resilience thinking offers possibilities to shift from control to prevention of zoonotic diseases in animal husbandry. The production of cheap animal protein at minimum costs has compromised animal health and increased zoonotic disease risks (Kimman, Hoek, & de Jong, 2013). In the light of climate change and food security, Ge et al. (2016) concluded that our focus on maximizing production has increased the vulnerability of production systems. Resilience thinking addresses change, adaptability and transformability on different levels (animal, farm and socio‐economical) which can lead to a more sustainable animal husbandry. Consequently, human and environmental health will benefit from this.

## 7 IMPLICATIONS FOR ONE HEALTH POLICIES IN ZOONOTIC DISEASE CONTROL

To justify zoonotic disease control measures like the culling of healthy animals, professional health workers and policy makers should make their underlying moral presuppositions about the moral status of animals more explicit, this could for example be achieved through the involvement of ethical expertise in expert committees that advice responsible authorities. This could contribute to more transparency of policy choices and acceptance of certain disease control measures by society. In case of moral dilemmas, it can be useful to apply VanDeVeer’s principle of two factor egalitarianism. This means that peripheral human interests are not accepted as a sufficient reason to take zoonotic disease control measures that seriously harm basis interests of animals. Moreover, these measures should not negatively affect long term resilience of animals and ecosystems. To improve further awareness, we suggest to emphasize the importance of ethical reflection on the outcome and consequences of One Health studies in standards like COHERE.

In our opinion, the goal of One Health should be to strive for a relatively stable equilibrium in which the health of humans, animals and the environment can be characterised as resilient. Moreover, if we try to understand the underlying mechanisms of resilience this will provide us opportunities to improve the health of humans, animals and the environment by means of prevention rather than cure. This is no easy task in the light of human dominance over animals and ecosystems. Even if you attribute humans a special moral status, the One Health concept, interpreted seriously, will define borders. Sufficient space for animals and the environment implies less room for humans to use animals and the environment only as resources.

## CONFLICT OF INTEREST

The authors have no conflict of interests to declare.
